# Detection Sensitivity Enhancement of Naphthalimide PET Fluorescent Probes by 4-Methoxy-Substitution

**DOI:** 10.3390/molecules25194465

**Published:** 2020-09-29

**Authors:** Ye Tian, Miao Li, Ying Liu

**Affiliations:** 1 College of Marine Technology and Environment, Dalian Ocean University, Dalian 116023, China; yingliu@dlou.edu.cn; 2 Key Laboratory of Environment Controlled Aquaculture, Ministry of Education, Dalian 116023, China; 3 School of Biological Engineering, Dalian Polytechnic University, Dalian 116023, China

**Keywords:** 1,8-naphthalimide, detection sensitivity, fluorescence quantum yield, molar extinction coefficient, photoinduced electron transfer

## Abstract

Naphthalimide photoinduced electron transfer (PET) fluorescent probes are widely used in fluorescence imaging. Thereinto, detection sensitivity is the vital parameter of PET probes. However, the modulation of detection sensitivity is yet to be reported for naphthalimide PET probes. Herein, the detection sensitivity enhancement of naphthalimide PET fluorescent probes through 4-methoxy-substitution is proposed in this work. Taking Zn^2+^ detection an example, 4-methoxy-naphthalimide PET probe 2-(2-(bis(pyridin-2-ylmethyl)amino)ethyl)-6-methoxy-1*H*-benzo[*de*]isoquinoline-1,3(2*H*)-dione (BPNM) and control PET probe 2-(2-(bis(pyridin-2-ylmethyl)amino)ethyl)-1*H*-benzo[de]isoquinoline-1,3(2*H*)-dione (BPN) are separately synthesized. The addition of 4-methoxy group with ability of strong electron donating to naphthalimide facilitates the construction of electronic push-pull system in the fluorophore resulting in the bathochromic shift of absorption and fluorescence emission spectra of BPNM and is further conducive to the enhancement of molar extinction coefficient ε and fluorescence quantum yield *Φ*_f_ of BPNM. Compared with BPN, BPNM shows lower Zn^2+^ detection limit in titration assays. Meanwhile, the fluorescence signal change (off-on) before and after Zn^2+^ addition of intracellular BPNM is more obvious and easier to control in confocal laser scanning imaging. Therefore, 4-methoxy-substitution improves the detection sensitivity of naphthalimide PET probe, which is favorable for the precise sensing of analyte, and further lays a good foundation for the synthesis of PET probe with high sensitivity.

## 1. Introduction

Fluorescence imaging is a sensor technique with non-invasive characteristics, high sensitivity, and excellent spatial and temporal resolution [1,2]. Fluorescence probe molecular devices, which are specially designed for analysis and detection purposes, have been widely used in the fields of biological, environmental, and medical testing, such as the detection of active small molecules, over-expressed enzymes and proteins in certain diseases, and intracellular micro-environment parameters [3,4].

The design of fluorescence probes can be realized through the utilization of different fluorescence detection mechanisms based on various detection environments and purposes. The development and understanding of fluorescence mechanisms promote a continuous, wider, and more flexible application of the fluorescence probe in the microscopic area. Fluorescence probes with large Stokes shift designed according to fluorescence resonance energy transfer (FRET) mechanism can generate accurate fluorescence ratiometric detection without the need for calibration [5,6,7]. Probes designed based on intra-molecular charge transfer (ICT) can realize small molecule long wavelength fluorescence emission, and detection of the micro-environment such as pH value and solvent polarity [8,9,10,11,12]. Meanwhile, the probes produced based on photo-induced electron transfer (PET) can realize remarkable fluorescence off-on change before and after detection [13,14,15,16,17]. A typical PET fluorescence probe is composed of a fluorophore conjugated with a recognition receptor via a short spacer. The PET process can be divided into a-PET and d-PET process, which correspond to the different transition directions of the electrons among the fluorophore and analyte recognition receptor. In the a-PET process, electron transfers from the recognition receptor to the fluorophore, because the highest occupied molecular orbital (HOMO) energy level (*E*_HOMO_) of the receptor is higher in magnitude than that of fluorophore. During d-PET, the excited state of the fluorophore donates electron to the lowest unoccupied molecular orbital (LUMO) of receptor and quenches fluorescence [18,19]. Thereinto, a-PET process is a more commonly used PET process.

To date, several fluorophores, including naphthalimide, cyanine dyes, and fluoroborate pyrrole, have been successfully applied to the design and preparation of fluorescence probes. 1,8-naphthalimide fluorophore has attracted extensive attentions due to its ease of structural modification, low cytotoxicity, high photo stability, pH stability, and two-photon absorption characteristics [20,21,22,23]. Specifically, the photophysical properties and ease of structural modification provide desirable diversity for the design and synthesis of naphthalimide fluorescence probes. Considering that the imine group of naphthalimide can be used as the condensing site to connect the fluorescence probe and electron-rich recognition group, naphthalimide is widely used as the fluorophore of a-PET fluorescence probe. When the recognition group of the probe is in the free state, the electrons transit from the HOMO of the recognition receptor to the HOMO of the naphthalimide fluorophore, quenching fluorescence of naphthalimide. On the contrary, when the analyte is combined with the recognition receptor, PET process is restrained and the fluorescence is recovered. Fluorescence probes require high sensitivity for improving detection efficiency. However, the improvement of the naphthalimide PET fluorescence probe sensitivity has yet to be reported.

The detection sensitivity of the fluorescence probe is closely related to the rate of change of the fluorescence signal with concentration [17]. The improvement of the fluorescence intensity of PET probe after PET is restrained can promote the detection sensitivity of probe under the constant probe concentration. The fluorescence intensity of probe at the specific concentration corresponds to two physical parameters; fluorescence quantum yield *Φ*_f_ and molar extinction coefficient ε [24]. Therefore, fluorophore with larger *Φ*_f_ and *ε* has higher detection sensitivity. The C4-substitution of naphthalimide with electron donating group is favorable for the construction of fluorophore electronic push-and-pull systems, which decreases the energy level difference between the fluorophore HOMO and LUMO and increases the *Φ*_f_ and ε. Therefore, C4-substitution with electron donating group can improve detection sensitivity of the naphthalimide fluorophore under the same experimental conditions. Usually, C4-substitution of naphthalimide mainly derives from the nucleophilic reaction of secondary or tertiary amine as the electron-donating group [21,22,23]. However, nitrogen atoms are prone to protonation, which weakens the electron donating ability of the amine groups, disturbs the original fluorophore electronic push-and-pull system, changes the fluorophore’s physical properties, and influences the detection performance of PET fluorescence probe. In contrast, strong electron donating groups, such as methoxy, are not easily influenced by the environment. Hence, C4-substitution with methoxy is more suitable for improving the detection sensitivity of naphthalimide PET probe.

**Scheme 1:**
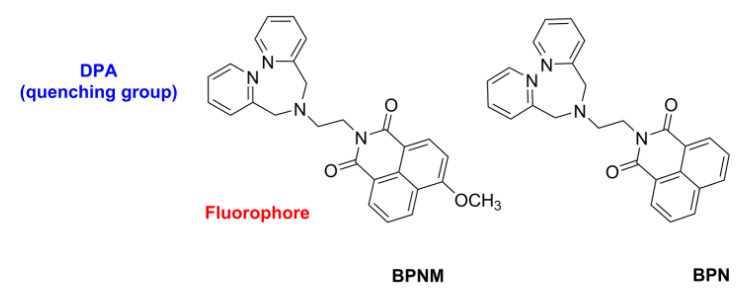
Structures of BPNM and BPN.

Zinc ions as the second most abundant transition metal ions in body and important metal ions in living organism, participate in various biological processes, such as apoptosis, gene expression, enzyme regulation, and neurotransmission [25]. Taking Zn^2+^ detection as an example, Zn^2+^ PET probe BPNM and unsubstituted Zn^2+^ naphthalimide PET probe BPN as the control were simultaneously synthesized to obtain the influence of C4-substitution with methoxy on detection sensitivity (Scheme 1). After Zn^2+^ recognition by BPNM (or BPN), the enhancement of fluorescence quantum yields indicated that the PET process is inhibited and BPNM (or BPN) can detect Zn^2+^ through monitoring fluorescence enhancement. Moreover, fluorometric titration testing showed that BPNM has lower detection limit compared to BPN. C4-substitution of naphthalimide with methoxy improving the PET probe detection sensitivity were further confirmed by confocal laser scanning microscope imaging; when BPNM was used for Zn^2+^ detection in cells, the signal off-on change was more substantial compared with BPN under the same detection conditions. Therefore, under the same detection conditions, C4-substitution with methoxy significantly improves detection sensitivity of naphthalimide PET probe, promoting the precise sensing of analyte and design of the PET fluorescence probe with high sensitivity.

## 2. Results and Discussion

### 2.1. 4-Methoxy Substitution of Naphthalimide

The development of C4-substitution of naphthalimide with methoxy is lacking due to the difficulty in modifying the C4 position. The only reported cases of such preparations are as follows: 4-methoxy-1,8-naphthalene dimethyl anhydride was synthesized via the reaction of 1,8-naphthalene dimethyl anhydride and sodium methylate in pyridine under iodide copper catalyzed conditions [26]. However, the method mentioned above possess some shortcomings, including that pyridine as solution is irritant and high toxicity; the iodide copper catalyst has a small particle size, making it difficult to separate from the final product by filtration and column chromatography.

Preparation of 4-methoxy-substituted naphthalimide can also be achieved via the reaction of 4-bromo naphthalimide and sodium methylate in methanol using anhydrous cupric sulfate as the catalyst [27]. The anhydrous cupric sulfate dissolved in methanol catalyzing the 4-Br-substituted naphthalimide replaced the original synthetic method of iodine copper catalyzing 1,8-naphthalene dimethyl anhydride in pyridine to undergo the reaction of C4-substitution of naphthalimide with methoxy. This modified method has the advantages of good water solubility and easy separation of the catalyst. Therefore, 4-methoxy-substituted Zn^2+^ PET probe BPNM using 2,2’-dipicolylamine (DPA) for molecular recognition were synthesized under these conditions. The corresponding synthetic route and compound characterization are shown in Scheme S1 and Figures S1–S10. Probes BPNM and BPN were successfully synthesized for further Zn^2+^ detection in the solutions and biological micro-environment.

### 2.2. Zn^2+^ Titration Experiments of BPNM and BPN

**Figure 1:**
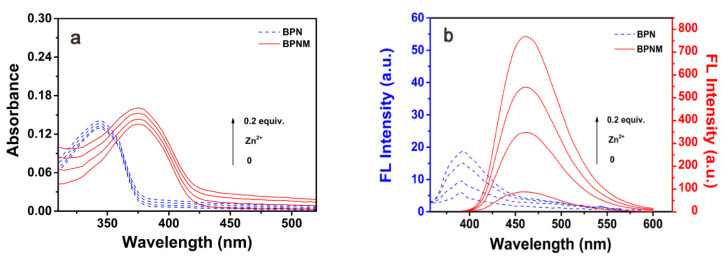
BPNM and BPN response spectra to Zn^2+^. (**a**) Absorption spectra and (**b**) fluorescence spectra of BPN (10 μM, λ_ex_ = 345 nm) and BPNM (10 μM, λ_ex_ = 379 nm) with different Zn^2+^ concentrations in the mixture of Tris-HCl (10 mM, pH = 7.4) and DMSO (*V*:*V* = 9:1).

In Figure S11, we can know that the HOMO energy levels of the BPNM and BPN fluorophore is lower than that of free receptors (DPA), but higher than that of zinc ion-combined receptors (DPA-Zn^2+^). Hence, in the free state of DPA, the PET process can originate in BPNM and BPN probes, where fluorophore emission is quenched. On the contrary, the PET is restrained and fluorescence light up after DPA coordination with zinc ions. The addition of BPNM and BPN to Tris-HCl solution (10 mM, pH = 7.4) and dimethyl sulfoxide (DMSO) (*V*:*V* = 9:1) solution generated varying degrees of response to Zn^2+^ in certain range of concentration, which is evident from the comparison between the absorbance (or emission spectra) of BPNM and BPN (Figure 1a,b). The comparison of BPNM and BPN spectra showed that the maximum absorption peak of BPNM had an obvious red shift relative to BPN (Figure 1a). Moreover, under the same reactant concentrations, the absorbance of BPNM showed a greater increase than that of BPN (Figure 1a). Similar results were observed in the fluorescence emission spectra of BPNM, which possessed a red shift compared to BPN (Figure 1b). Additionally, the BPNM displayed a 2-fold increased fluorescence intensity compared to BPN (Figure 1b). By comparing chemical structure, it can be concluded that the differences between BPNM and BPN spectra mainly originate from the 4-methoxy substitution of naphthalimide. The strong electron donating methoxy group acting as the auxochromic group, which enhances the fluorophore electron push-and-pull, connected to the naphthalimide fluorophore, reducing the energy level difference between the fluorophore HOMO and LUMO (from 4.01 to 3.79 eV) with the red-shift of the maximum absorbance peak and fluorescence emission peak (Figure 1 and Figure S11). The molar extinction coefficients of BPNM and BPN for Zn^2+^ recognition are 1.617 × 10^5^ and 1.407 × 10^5^ L·Mol^−1^·cm^−1^, where the value of BPNM showed a significant increases compared to BPN. Furthermore, the *Φ*_f_ of BPNM and BPN before and after Zn^2+^ recognition are shown in Table 1. Under constant probe concentration conditions, after the addition of a specific amount of Zn^2+^, the *Φ*_f_ of BPNM (0.628) is considerably higher than that of BPN (0.014). In summary, both BPNM and BPN can be used as PET probe for fluorescence detection of Zn^2+^. Additionally, incorporation of the 4-methoxy group provides the naphthalimide PET probe with higher molar extinction coefficient and fluorescence quantum yield. 

### 2.3. Determination and Comparison of Limit of Detection (LOD) of BPNM and BPN Probes

**Figure 2:**
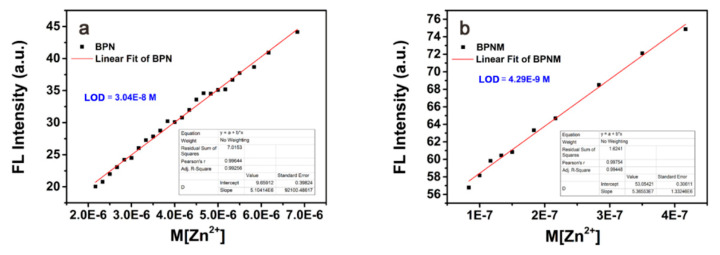
Determination of (**a**) BPN and (**b**) BPNM detection limit.

The detection sensitivity of the PET fluorescence probe was quantitatively analyzed by LOD. Zn^2+^ concentration in the Tris-HCl (10 mM, pH = 7) and DMSO (*V*:*V* = 9:1) solutions was plotted as the abscissa and the fluorescence intensity of the probe after Zn^2+^ was plotted as the ordinate. As shown in Figure 2, within a certain Zn^2+^ concentration range, the fluorescence intensities of BPNM and BPN are linearly correlated to the Zn^2+^ concentration, in which the slope fitted is *k*. *σ* value is the standard deviation value of the three measurements of the fluorescence intensity without the addition of Zn^2+^. The probe LOD can be calculated using Equation (1):

(1) Detection limit=3 σ/κ

### 2.4. BPNM and BPN Ion Competitive Experiment

**Figure 3:**
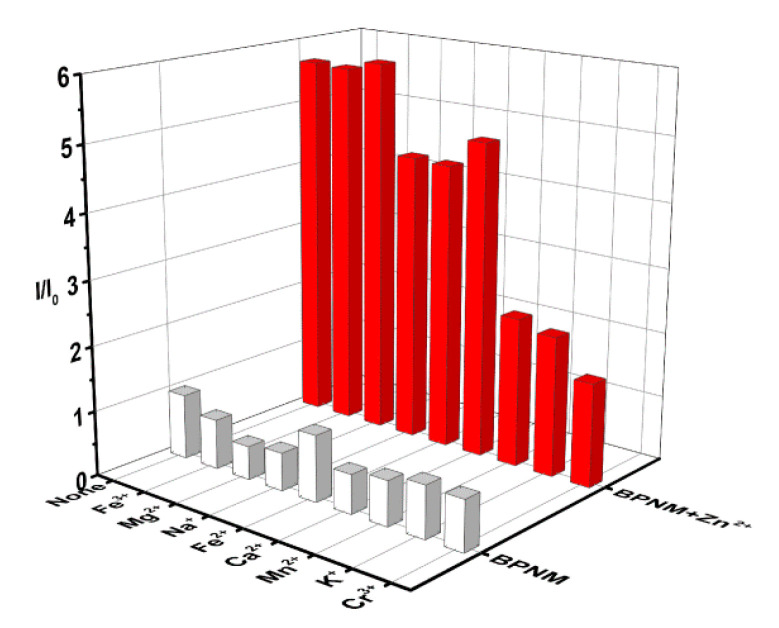
Fluorescence intensity (*I*) responding to other metal ions of nitrates (30 μM) with zinc ions (30 μM) existed or not. *I*_0_ is the fluorescence intensity of BPNM (30 μM).

In the Tris-HCl solution (10 mM, pH = 7) and DMSO (*V*:*V* = 9:1) system, the fluorescence intensity of 10 μM BPNM (or BPN) was determined as *I_0_*. A variety of metal ions such as Na^+^, Fe^2+^, Fe^3+^, Mg^2+^, Mn^2+^, Cr^3+^, K^+^, or Ca^2+^ (30 μM, nitrates), were added into the system, where no new fluorescence peaks were observed for BPNM (or BPN), and the *I*/*I_0_* value was approx 1 (Figure 3 and Figure S12). However, when Zn^2+^ (30 μM) was continuously added into the system, the fluorescence intensity of BPNM (or BPN) increased (Figure 3 and Figure S12). Therefore, in the presence of other metal ions, BPNM (or BPN) can selectively recognize Zn^2+^ with the obvious increase of fluorescence intensity, and the intensity change after Zn^2+^ recognition is simple to monitor.

### 2.5. BPNM and BPN Cytotoxicity Analysis

**Figure 4:**
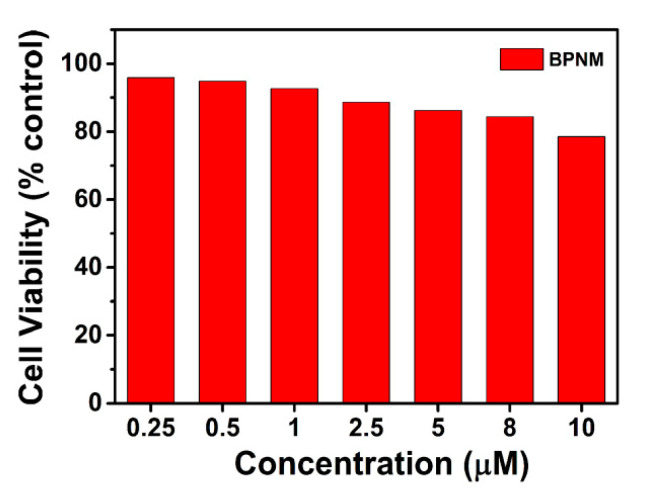
BPNM cytotoxicity analyzed by MTT assays at various concentrations (0.25, 0.5, 1, 2.5, 5.0, 8.0, and 10.0 μM) in living MCF-7 cells for 12 h.

The biocompatibility of BPNM (or BPN) was evaluated using 3-(4,5-dimethylthiazol-2-yl)-2,5*-*diphenyltetrazo-lium bromide (MTT) assay. The results showed that after incubation of BPNM (or BPN) (0, 0.25, 0.5, 1.0, 2.5, 5.0, 8.0, and 10.0 μM) with human breast cancer cells MCF-7 for 12 h, the cells displayed above 75% survival rate and high activity (Figure 4 and Figure S13). Hence, BPNM (or BPN) probe has good biocompatibility and low cytotoxicity, which can be further used as a fluorescence sensor for Zn^2+^ detection in biological micro-environment.

### 2.6. Confocal Laser Scanning Imaging of BPNM and BPN in Cells

**Figure 5:**
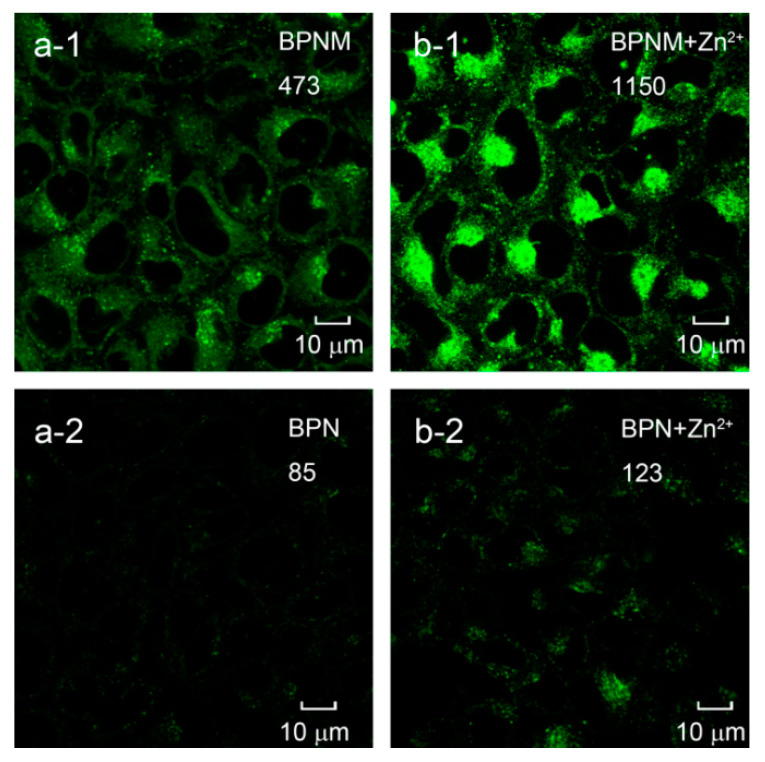
Two-photon confocal fluorescence images and average fluorescence intensity of MCF-7 cells stained with probe (1) BPNM and (2) BPN. Before imaging, (**a**) cells and BPNM (or BPN) (50 μM), (**b**) cells, BPNM (or BPN) (50 μM), and Zn^2+^ (50 μM) were co-incubated in culture medium.

The fluorescence signal can be detected by confocal laser scanning microscopy (CLSM). For cell passage, MCF-7 cells were vaccinated on the confocal cell culture dish at density of 10^5^/dish, followed by the addition of 10% fetal Dulbecco’s modified Eagle’s (DMEM) with bovine serum. The cell dish was placed in an incubator filled with 5% CO_2_ and 95% air at 37 °C and the adhesive cells were cultured for 24 h. The experimental steps of the intracellular BPNM probe imaging are as follows: BPNM probe (50 μM) was added into the confocal cell culture dish and incubated in the incubator for 20 min. The dish was removed from the incubator, the culture medium was disposed, the cells were rinsed 3 times (2 mL) with phosphate buffer (PBS, pH = 7.4, 0.01 μM), and new DMEM culture medium was subjected to confocal laser scanning imaging. The BPNM probe fluorescence was excited by a two-photon laser at 800 nm wavelength using a titanium-sapphire lock mode laser (Maitai, Spectra-Physics) and collected by RXD3 channel at 380–560 nm wavelength. The inverted cell fluorescence images were imaged by the laser confocal scanning microscope (Olympus FV1000) and the results were further post-treated and analyzed by Olympus FV10-ASW 3.0 software. The regions of interest (ROI) were selected on the cell fluorescence image, in which the average fluorescence intensity of the cell was statistically analyzed and the average fluorescence intensity of the cell in the above experiment was calculated as 473 in the ROI (Figure 5). After cell fluorescence imaging of BPNM, Zn^2+^ (1 equiv.) was added to the above culture dish and mixed with the culture medium, the cells were placed in the incubator for a further 20 min. Then, the original culture medium was disposed and the new DMEM culture medium was imaged on the confocal microscope. After fluorescence imaging, the average fluorescence intensity of the BPNM with Zn^2+^ addition in the same ROI was calculated and the average fluorescence intensity of the cell after Zn^2+^ addition was calculated as 1150 (Figure 5). Furthermore, the fluorescence imaging of the MCF-7 cell dyed by BPN (50 μM) was conducted in the same manner. After imaging, the average fluorescence intensity of BPN before and after the addition of Zn^2+^ (1 equiv.) calculated by the above calculating method of fluorescence intensity were 85 and 123, respectively (Figure 5). 

The results show that the fluorescence intensity change of BPNM probe for Zn^2+^ recognition is more substantial than that of BPN. As shown in Figure S14, we can know that the TPA cross section of BPNM is about one time larger than TPA cross section of BPN. Moreover, the change rate of BPNM fluorescence signal with Zn^2+^ concentration is much larger than that of BPN. Considering that the change rate of probe fluorescence intensity with analyte concentration corresponds to TPA cross section, which is equivalent to the role of molar extinction coefficient in one-photon fluorescence imaging, and fluorescence quantum yield in two-photon fluorescence imaging. Hence, it can be concluded that C4-substitution with methoxy group simultaneously increases TPA cross section and molar extinction coefficient of naphthalimide PET probe, improving that the detection sensitivity of naphthalimide PET probe BPNM.

## 3. Materials and Methods

### 3.1. Materials and Instrument

The reagent grade solvents and reagents used in the experiment were purchased from Shanghai Sahn chemical technology Co., Ltd. The experimental water was taken from Milli-Q ultrapure water system. When preparing the experiment, BPNM and BPN dissolved in DMSO (20 mM) was stored in a refrigerator at −20 °C for use. ^1^H NMR and ^13^C NMR spectra of BPNM and BPN were measured by Bruker Avance II 400 MHz nuclear magnetic resonance spectrometer (Madison, WI, USA). Meanwhile, the mass spectra of BPNM and BPN are measured by Agilent LC/Q-TOF mass spectrometer (Palo Alto, CA, USA). The fluorescence emission and ultraviolet-visible absorbance spectra were separately measured by Agilent Technologies Cary Eclipse fluorescence spectrophotometer (Palo Alto, CA, USA) and Agilent Technologies Cary 60 UV-Vis spectrophotometer (Palo Alto, CA, USA) separately. In order to adjust the fluorescence intensity within the appropriate range, the excitation and emission slit width of the fluorescence spectrometer was 5/5 nm respectively. The silica gel used in the fast column chromatography method was 200–300 mesh, purchased from Qingdao Ocean Chemical Limited company.

### 3.2. Methods

#### 3.2.1. BPNM and BPN Synthesis Method

The synthetic methods of BPN, BPNM, and its intermediate compounds **1–4** have been described in reference [27]. The ^1^H NMR spectra of BPN, BPNM, and compounds **1**–**4**, ^13^C NMR and HRMS spectra of BPN and BPNM are shown in Supporting Information.

#### 3.2.2. Fluorescence Quantum Yield Determination

The absolute fluorescence quantum yield was determined by the absolute quantum yield instrument and the testing steps are as follows: Firstly, the absorbance wavelength of the probe in testing solvent was inputted, the solvent background was corrected by running only the solvent. Next, the sample transmittance was tested by loading the probe sample at 1 × 10^−4^ M concentration. The fluorescence quantum yield (*Φ*_f_) was defined as the ratio between the emission photon number of probe molecule (*PN*_em_) and the absorbance photo number of probe molecule (*PN*_abs_) (Equation (2)). The absorbance photo number of probe molecule (*PN*_abs_) was obtained through subtracting the transmittance photon number (*PN*_trans_) from the total photon number (*PN*_all_).

(2) $$\Phi_{f}=PN_{\mathrm{en}}/PN_{\mathrm{abs}}=PN_{\mathrm{en}}/\left(PN_{\mathrm{all}}-PN_{\mathrm{trans}}\right)$$

#### 3.2.3. Cell Culture and MTT Experiment

Human breast cancer MCF-7 cells were commercially supplied by KeyGEN BioTECH Co., Ltd. (Nanjing, Jiangsu, China) The culture medium was the Dulbecco’s modified Eagle’s culture medium containing 10% fetal bovine serum and 1% streptomycin-penicillin, and the culture conditions were the adherent culture in an incubator filled with the mixture of 5% CO_2_ and 95% air. The cell activity was determined and evaluated by MTT experiment. 

Mitochondrial dehydrogenases in the mitochondria of the living cells has the ability to restore 3-(4,5-dimethylthiazol-2-yl)-2,5*-*diphenyl tetrazolium bromide (MTT) in water-insoluble blue purple formazan, while the dead cells do not have this ability. Within a certain cell counting range, the amount of formazan crystalline formation amount was proportional to the living cell amount. The formazan crystalline deposited in the cell was dissolved in DMSO and its absorbance was determined by microplate reader instrument at specific wavelength. The cells not cultured by probe were used as the negative control group, and by calculating the ratio between the absorbances of the probe cultured group and negative control group, the percentage of cell activity under the culture conditions was obtained (cell activity of the negative control group: 100%). 

Microplate reader required the utilization of 96-hole plate for MTT assays. Therefore, after the cells were digested, 100 μL culture medium containing MCF-7 cells and 10% fetal bovine serum was added to the 96-hole plate (Costar) for cell passage. In order to ensure data effectiveness, the cell inoculum density in each hole should reach 1 × 10^5^ /mL. After cell adhesion for 24 h, the original culture medium was disposed and the 96-hole plate was rinsed with phosphate buffered saline (PBS, 0.01 M, pH = 7.4) (100 μL/hole). Then, every 6 multiple holes were divided as a group of parallel experiment, the cells in each group were cultured by the complete culture medium containing 0, 0.25, 0.5, 1, 2, 5, 8 or 10 μM BPMN. The results of the experimental and control groups were statistically obtained by each parallel experiment containing 6 multiple pores. After being cell-cultured for 12 h, PBS solution of MTT was added into the 96-pore plate (10 μL/pore, 5 mg/mL), which was placed in the cell incubator for 4 h. Then the culture medium was removed and the formazan deposited at the bottom of the cell was dissolved in DMSO (100 μL/pore). Finally, the microplate reader was used to measure the absorbances at 490 and 570 nm wavelengths by calculating the cell activity according to Equation (3).

(3) $$\mathrm{Cell}\;\mathrm{activity}(\%)=\frac{OD_{490}{{}_{nm\;}}_{Probe}-OD_{570nm\;}{}_{Probe}}{OD_{490nm\;Control}-OD_{570nm\;Control}}\times 100\%$$

## 4. Conclusions

Naphthalimide is commonly used as a PET fluorescence probe for biological micro-environment detection, but requires high detection sensitivity to be effective. Therefore, the method for improving the sensitivity of the naphthalimide PET fluorescence probe is worth exploring. In this work, the 4-methoxy-naphthalimide PET probe BPNM and control naphthalimide PET probe BPN for Zn^2+^ detection were simultaneously synthesized to compare the influence of 4- methoxy substitution on detection sensitivity of naphthalimide PET probe. 4-methoxy-naphthalimide PET probe BPNM was prepared using anhydrous cupric sulfate as the catalysis in methanol, while PET probe BPN without 4-Methoxy substitution were synthesized as the control molecular. By comparing the absorbance and fluorescence spectra, and analysis of HOMO-LUMO orbit energy between BPNM and BPN probes, it revealed that C4-substitution with methoxy of naphthalimide enhanced the electron push-and-pull of the fluorophore system, reduced the energy level difference between the HOMO and LUMO orbits of the fluorophore, promoted a red shift of the maximum absorbance and fluorescence emission peaks, and increased the molar extinction coefficient and fluorescence quantum yield of the probe. Furthermore, compared to BPN (3.04 × 10^−8^ M), BPNM (4.29 × 10^−9^ M) showed lower Zn^2+^ detection limit. Meanwhile, BPNM, which showed increased detection sensitivity, compared to BPN, as well as low cytotoxicity and specific Zn^2+^ recognition, can be used to recognize intracellular Zn^2+^. In intracellular microenvironment, BPNM exhibited more substantial fluorescence intensity change for Zn^2+^ recognition, compared to BPN. Hence, C4-substitution with methoxy provides a novel approach to improve the naphthalimide PET probe, which is favorable for precise study of intended analyte and allows for further design and synthesis of PET probes with high sensitivity.

## Figures and Tables

**Table 1:** Photophysical properties of BPNM and BPN.

	λ_abs_ (nm) ^1^	λ_em_ (nm) ^2^	*Φ*_f_^3^	*Φ*_f_^4^
**BPNM**	379	457	0.058	0.628
**BPN**	345	396	0.003	0.014

Wavelengths of ^1^ absorption peaks and ^2^ emission peaks excited by the corresponding excited wavelengths in 10 mM Tris-HCl buffer (pH = 7.4) and DMSO (*V*:*V* = 9:1) solution; fluorescence quantum yields of chemosensors (0.1 mM) ^3^ before and ^4^ after the addition of Zn^2+^ (0.1 mM) to 10 mM Tris-HCl buffer (pH = 7.4) and DMSO (*V*:*V* = 9:1) solution.

## Supplementary Materials

Scheme S1: Synthesis routes of probe BPN and BPNM, Figure S1: ^1^H NMR spectrum of compound **1** recorded in CDCl_3_, Figure S2: ^1^H NMR spectrum of compound **2** recorded in CDCl_3_, Figure S3: ^1^H NMR spectrum of compound **3** recorded in CDCl_3_, Figure S4: ^1^H NMR spectrum of compound **4** recorded in CDCl_3_, Figure S5: ^1^H NMR spectrum of compound **2** recorded in CDCl_3_, Figure S6: ^1^H NMR spectrum of compound **6** recorded in CDCl_3_, Figure S7: ^1^H NMR spectrum of compound BPNM recorded in CDCl_3_, Figure S8: ^13^C NMR spectrum of compound BPNM recorded in CDCl_3_, Figure S9: Q-TOF mass spectrum of compound BPN, Figure S10: Q-TOF mass spectrum of compound BPNM, Figure S11: (a) Molecular structures and illustrations of frontier molecular orbital energy and PET process between different states of receptor (DPA) and (b) BPNM or (c) BPN fluorophore moieties by B3LYP/ 6–31 g (d, p) Gaussian calculation, Figure S12: Fluorescence intensity (*I*) responding to other metal ions of nitrates (30 μM) with zinc ions (30 μM) existed or not. *I*_0_ is the fluorescence intensity of BPN (30 μM), Figure S13: BPN cytotoxicity analyzed by MTT assays at various concentrations (0.25, 0.5, 1, 2.5, 5.0, 8.0, and 10.0 μM) in living MCF-7 cells for 12 h, Figure S14: TPA spectra of BPNM (0.1 mM) and BPN (0.1 mM Tris-HCl 10 mM, 60 mM KCl, pH = 7.4).
