# Supplementary material for: Detection Sensitivity Enhancement of Naphthalimide PET Fluorescent Probes by 4-Methoxy-Substitution
Source: Molecules. 2020 Sep 29;25(19):4465. doi: 10.3390/molecules25194465 (PMC7582873; doi:10.3390/molecules25194465)
Supplement: Supplementary file 1 [file molecules-25-04465-s001.pdf]

Supplementary file 1

Detection sensitivity enhancement of naphthalimide PET fluorescent probes by 4-methoxy-substitution

Ye Tian [1,2],* Miao Li [3,*], and Ying Liu [1,2]

[1] College of Marine Technology and Environment, Dalian Ocean University, Dalian 116023, China; yingliu@dlou.edu.cn (Y.L.)
[2] Key Laboratory of Environment Controlled Aquaculture, Ministry of Education, Dalian 116023, China; yingliu@dlou.edu.cn (Y.L.)
[3] School of Biological Engineering, Dalian Polytechnic University, Dalian 116023, China;
* Correspondence: tianye@dlou.edu.cn (Y.T.); lim@mail.dlut.edu.cn (M.L.);
Tel.: +86-0411-8476-3255 (Y.T.); +86-0411-8632-2228 (M.L.)

1. Synthesis routes of probe BPN and BPNM

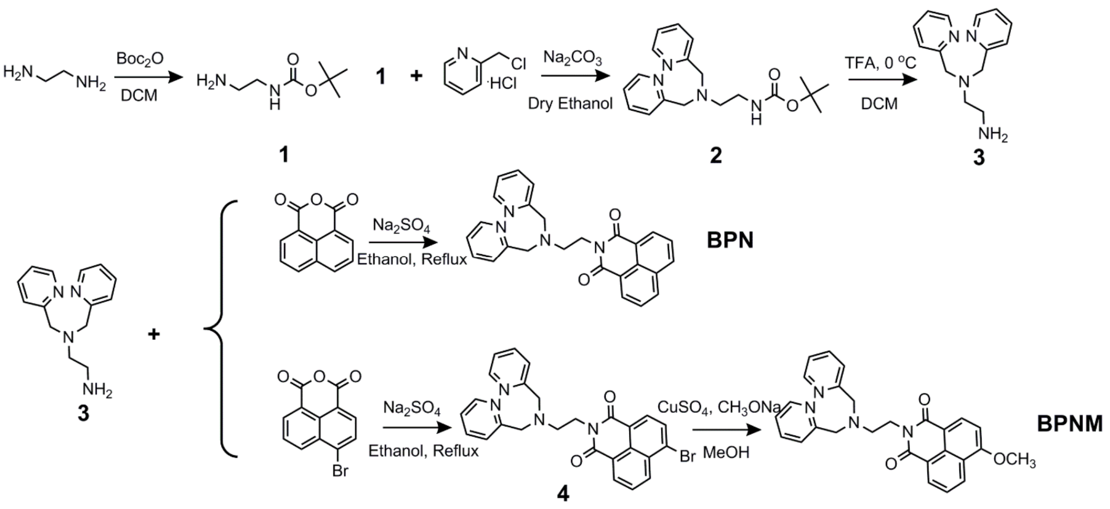

Scheme S1 Synthesis routes of probe BPN and BPNM

2. Synthesis method of BPNM, BPN, and intermediate products

2.1 **Synthesis of compound 1**

Under the condition of vigorous stirring at 0°C, Boc$_2$O (5.8 mL, 25.0 mM) dissolved (100 mL) in 100 mL of dichloromethane was slowly dropped into the dichloromethane solution (100 mL) of ethylenediamine (10.0 mL, 150 mM) within 3 h and the above mixture was stirred for 16 h. After the reaction, the solvent was evaporated, the oily residue was dissolved in 60 mL of sodium carbonate aqueous solution, and extracted with dichloromethane (2×60 mL). The organic layer was dried over anhydrous Na_2SO_4 and the solvent was evaporated under reduced pressure to yield compound **1** (a colorless viscous liquid, 1.44 g, 100%).

2.2 **Synthesis of compound 2**

Compound **1** (0.8 g, 5.0 mmol), sodium carbonate (2.4 g, 22.6 mmol) and 2- pyridylmethyl chloride hydrochloride (1.8 g, 10.9 mmol) were dissolved in

15 mL of absolute ethanol. The above mixture was refluxed for 12 h under nitrogen protection. After the reaction, the solvent was distilled under reduced pressure. The residue was dissolved in 50 mL of sodium hydroxide aqueous solution and extracted with dichloromethane (3×30 mL). Then the organic layer was separated, dried with potassium carbonate, and evaporated. The residue was separated by silica gel flash chromatography using the mixture of n-hexane: ethyl acetate: methyl alcohol (V_1: V_2: V_3= 140: 60: 7.5) as eluent to obtain compound **2** (1.5 g, 91%) as yellow viscous oil.

2.3 **Synthesis of compound 3**

At 0°C, compound **2** (130 mg, 0.39 mmol) dissolved in 8 mL of dichloromethane was added dropwise to 0.5 mL of trifluoroacetic acid solution. The mixture reacted at room temperature for 25 h. After the reaction, the solvent was evaporated. The residue was dissolved in sodium hydroxide aqueous solution and extracted with dichloromethane. The solvent was evaporated to obtain a yellow oil, compound **3** (85 mg, yield 90%).

2.4 **Synthesis of compound 4**

Compound **3** (52.5 mg, 0.217 mM) was dissolved by 5 mL of absolute ethanol in 10 mL round bottom flask,. After adding 4-bromo-1,8-naphthalic anhydride (56.76 mg, 0.205 mM) and a small amount of anhydrous Na_2SO_4, the reaction solution was refluxed for 6 h under the protection of nitrogen. After the reaction, the solution was evaporated and the residue was separated through silica gel flash chromatography. Thereinto, compound **4** (yellow oil)

was collected using the eluent composed of methane dichloride: methanol (V_1: V_2 = 40: 1). Compound **4** was obtained as yellow solid after vacuum drying (43 mg, yield 42%).

2.5 **Synthesis of probe BPN**

In a 10 mL round-bottom flask, compound **3** was dissolved in 5 mL of absolute ethanol. After adding 1,8-naphthalic anhydride (100 mg, 0.51 mM) and a small amount of anhydrous Na_2SO_4, the mixture solution refluxed for 6 h under nitrogen protection. After the reaction, the solvent was evaporated and the residue was further separated through silica gel flash chromatography. Thereinto, compound **BPN** (yellow oil) was collected using the eluent composed of methane dichloride: methanol (V_1: V_2 = 20: 1). Compound **BPN** was obtained as yellow solid after vacuum drying (72 mg, yield 33%).

Collect the methane dichloride: methanol (v: v = 20: 1) eluted fractions, and spin dry to get a yellow oil , Compound BPN (yellow solid after vacuum drying, 72 mg, yield 33%).

3. ^{1}H-NMR, ^{13}C-NMR, and TOF-MS spectra of BPN, BPNM, and intermediates.

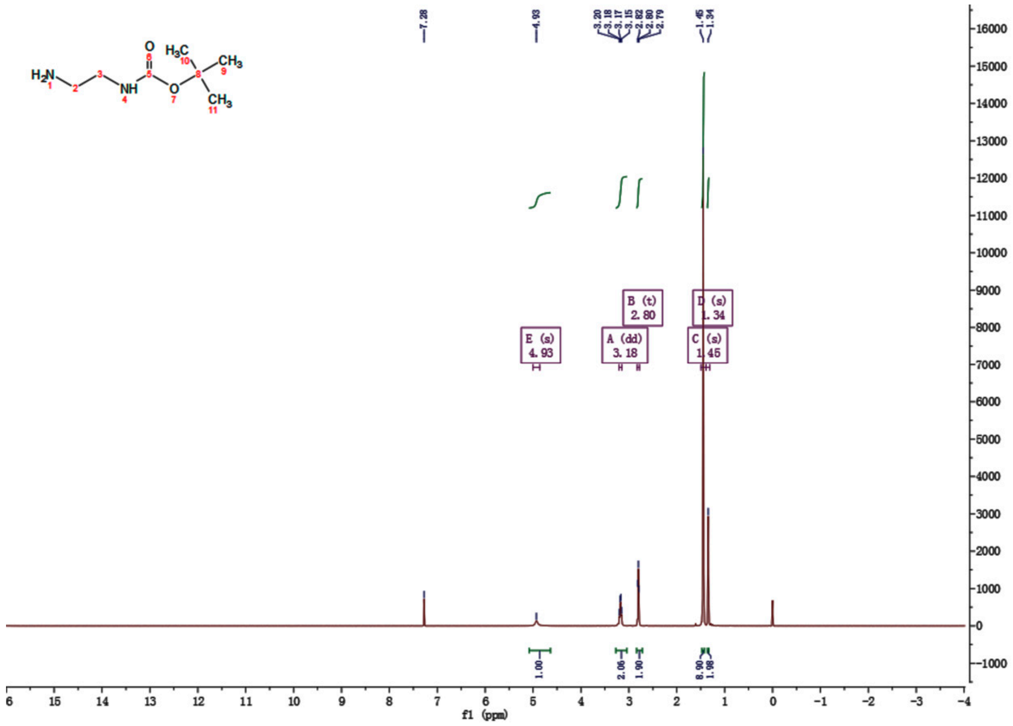

Figure S1 ^{1}H NMR spectrum of compound 1 recorded in $CDCl_3$

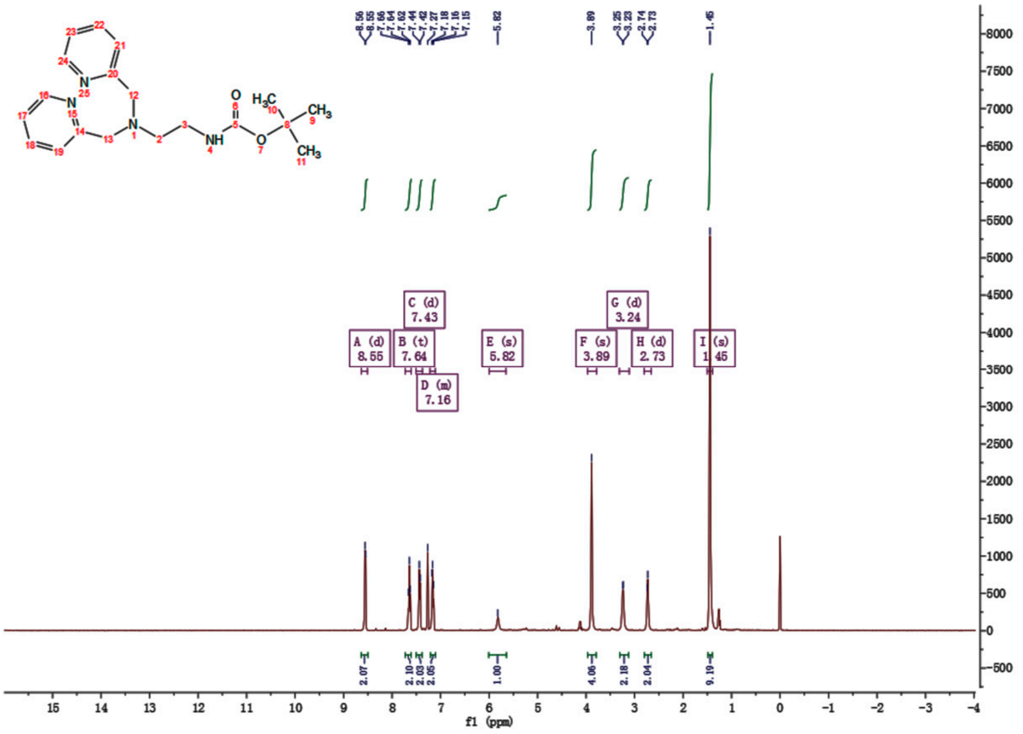

Figure S2 ^{1}H NMR spectrum of compound 2 recorded in $CDCl_3$.

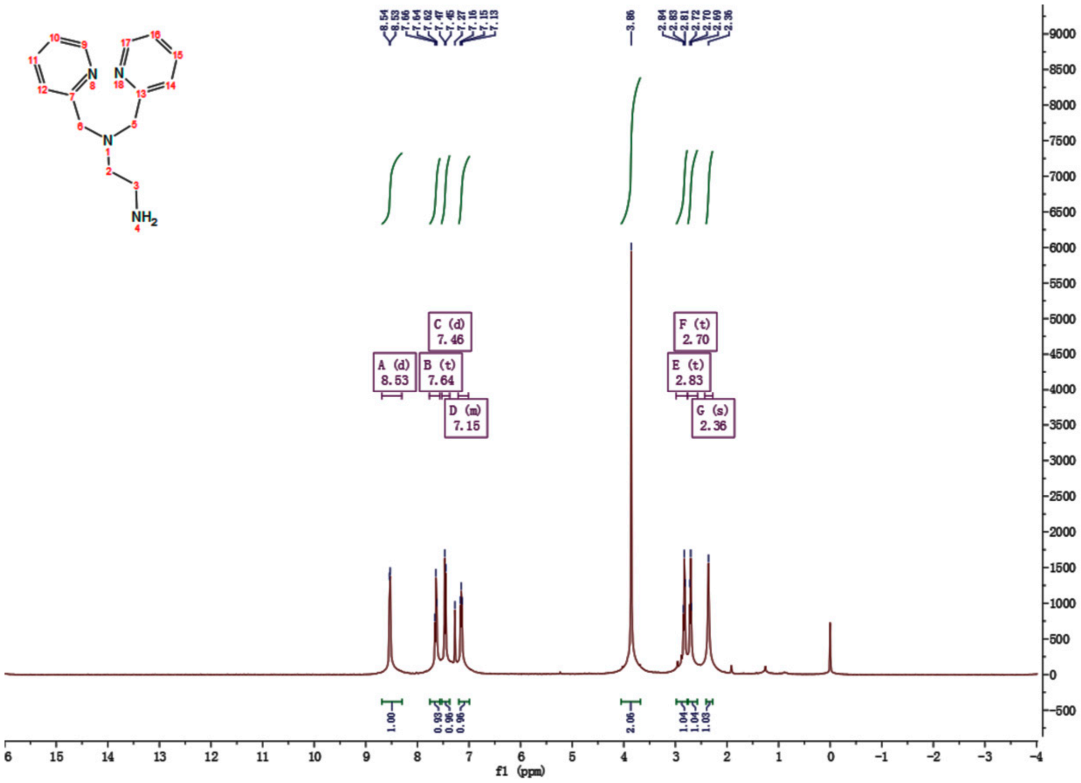

Figure S3 ^{1}H NMR spectrum of compound 3 recorded in $CDCl_3$.

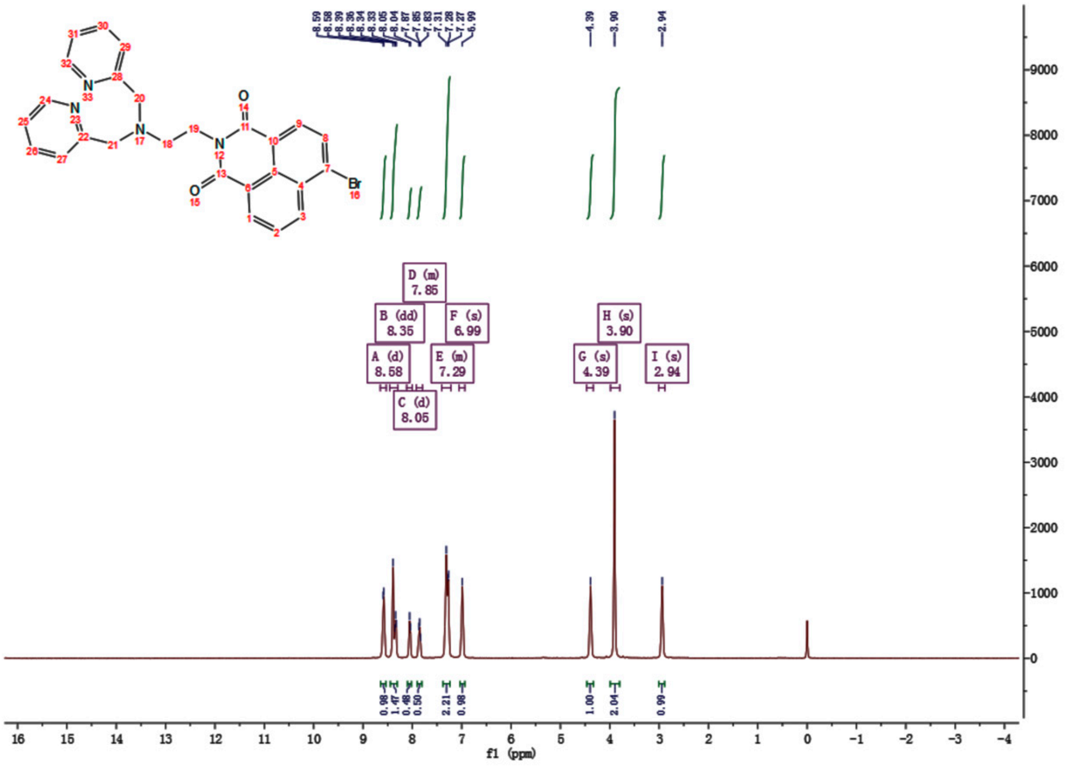

Figure S4 ^{1}H NMR spectrum of compound **4** recorded in $CDCl_3$.

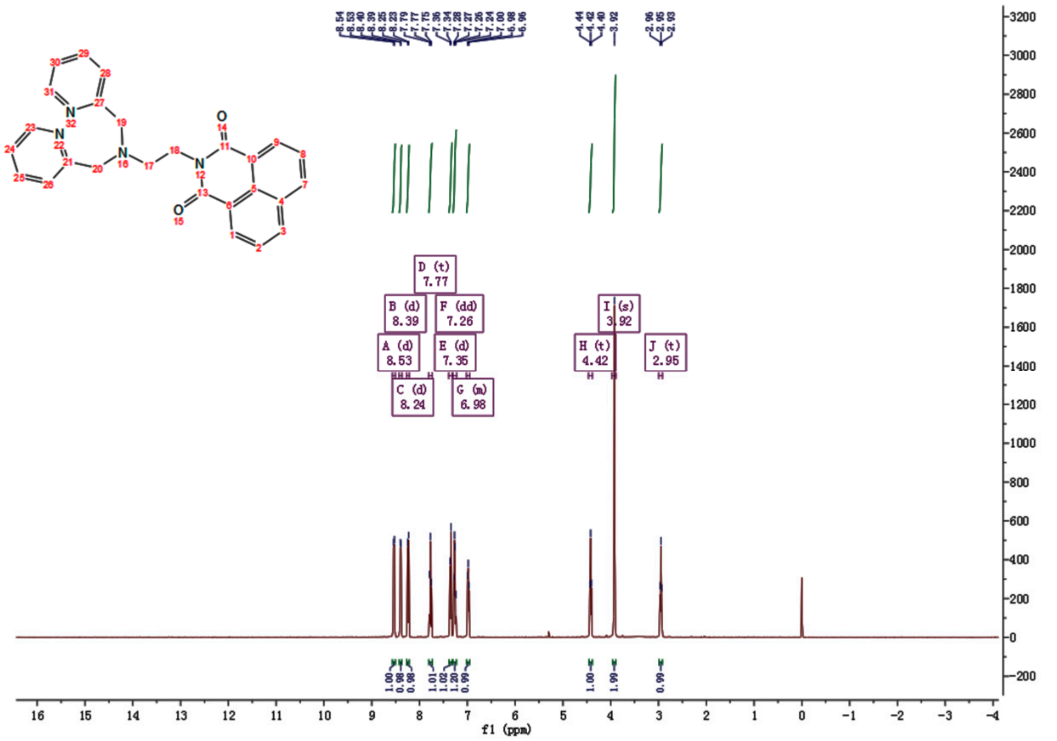

Figure S5 ^{1}H NMR spectrum of compound **2** recorded in CDCl3.

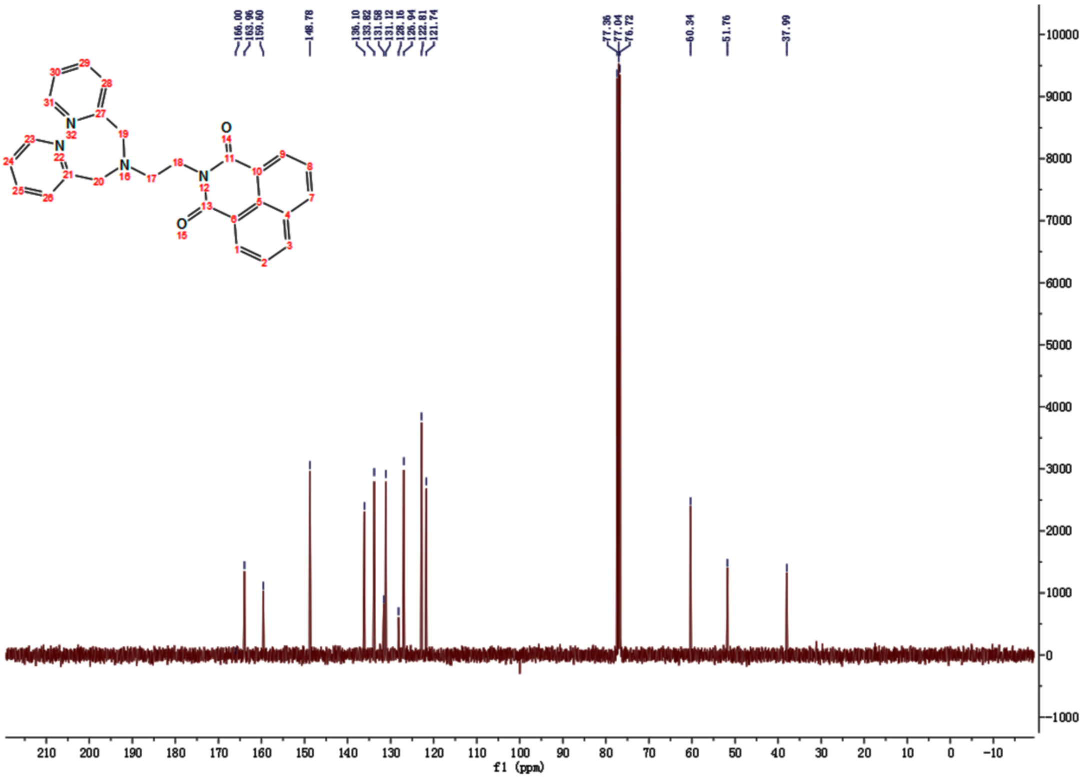

Figure S6 [1] H NMR spectrum of compound **6** recorded in CDCl3.

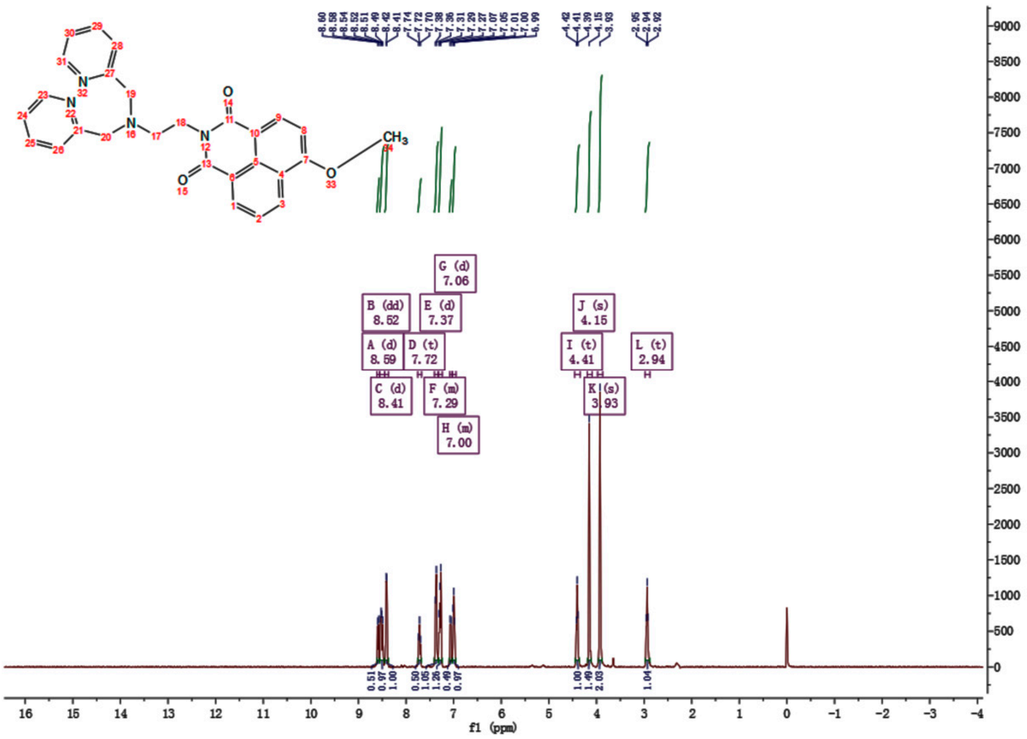

Figure S7 ^{1}H NMR spectrum of compound **BPNM** recorded in $CDCl_3$

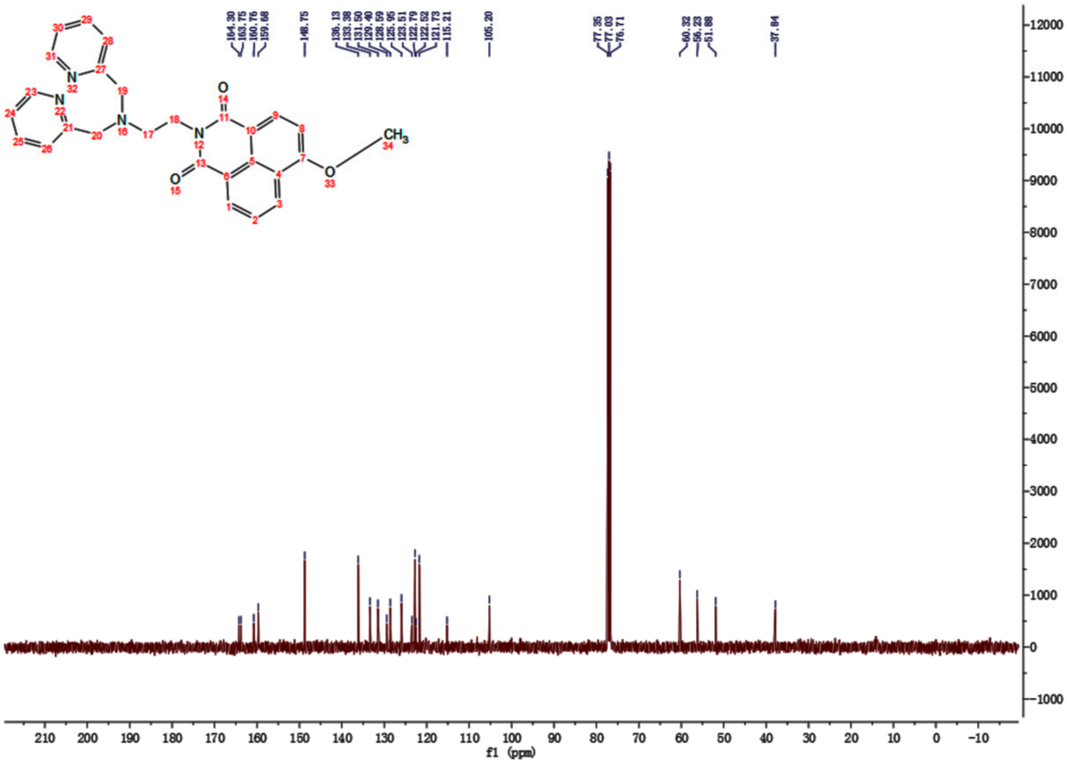

Figure S8 ^{13}C NMR spectrum of compound **BPNM** recorded in $CDCl_3$

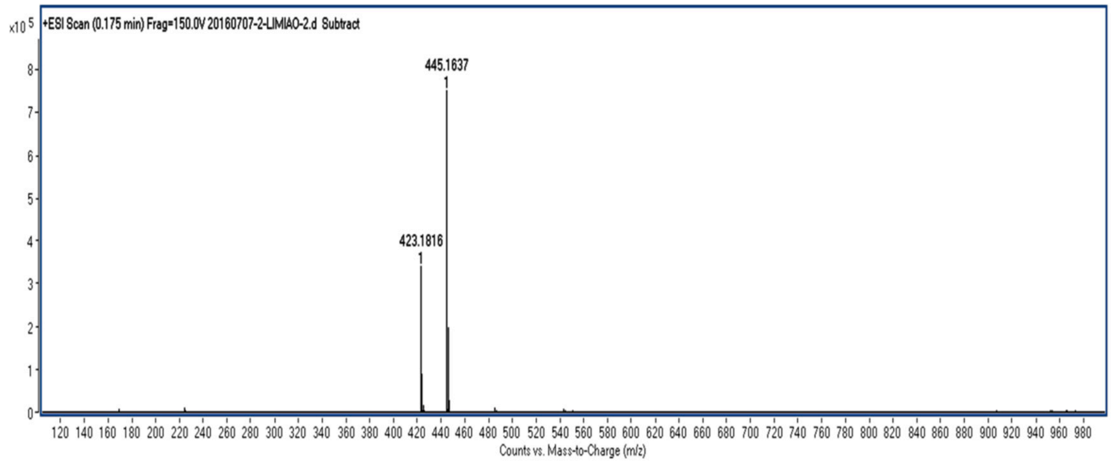

Figure S9 Q-TOF mass spectrum of compound **BPN**

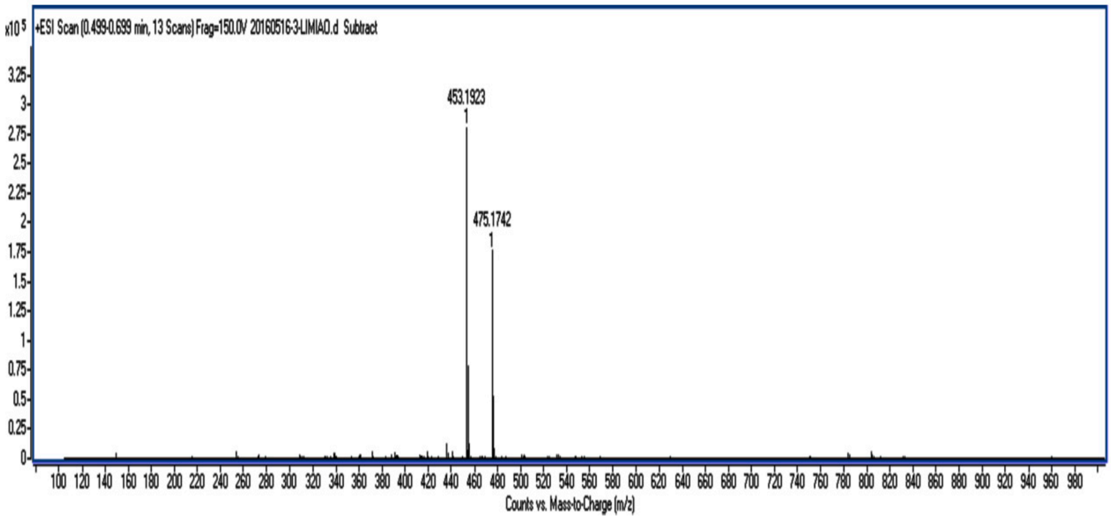

Figure S10 Q-TOF mass spectrum of compound BPNM

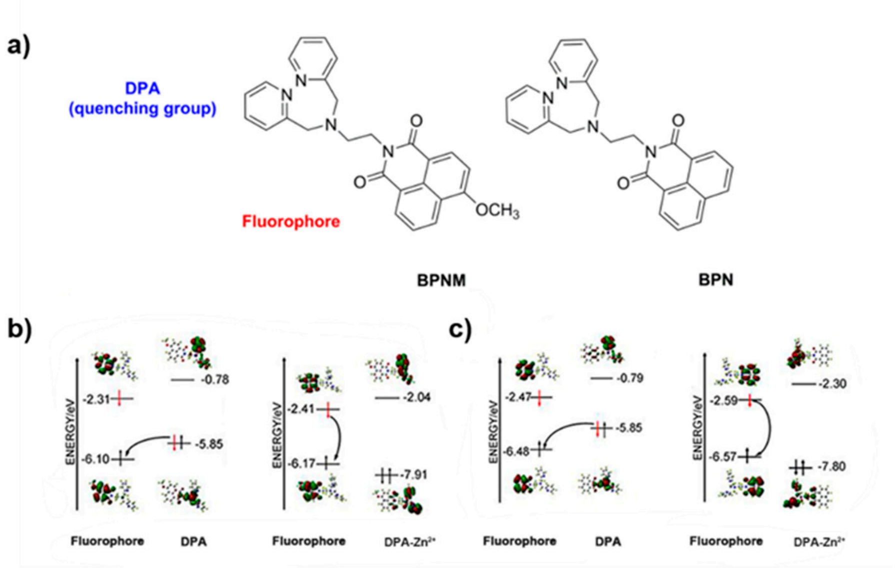

Figure S11 (a) Molecular structures of **BPNM** and **BPN**. Frontier molecular orbital energy illustrations of sensors (b) **BPNM** and (c) **BPN** and the PET process between different states of receptor (DPA) and **BPNM** (or **BPN**) fluorophore moieties by B3LYP/ 6-31g (d, p) calculation. (The geometries of the molecules at the ground state (S0) and the excited state (S1) were optimized by density functional theory (DFT) and time-dependent DFT (TD-DFT) methods with B3-LYP functional and 6–31G (d, p) basis set. No constraints to bonds/angles/dihedral angles were applied in the calculations and all atoms were free to optimize. The frontier molecular orbital energy of DPA, DPA-Zn^{2+}, and fluorophore moieties were studied by B3LYP/6–31 g (d, p) calculation. All theoretical calculations were carried out using the Gaussian 09 program package.)

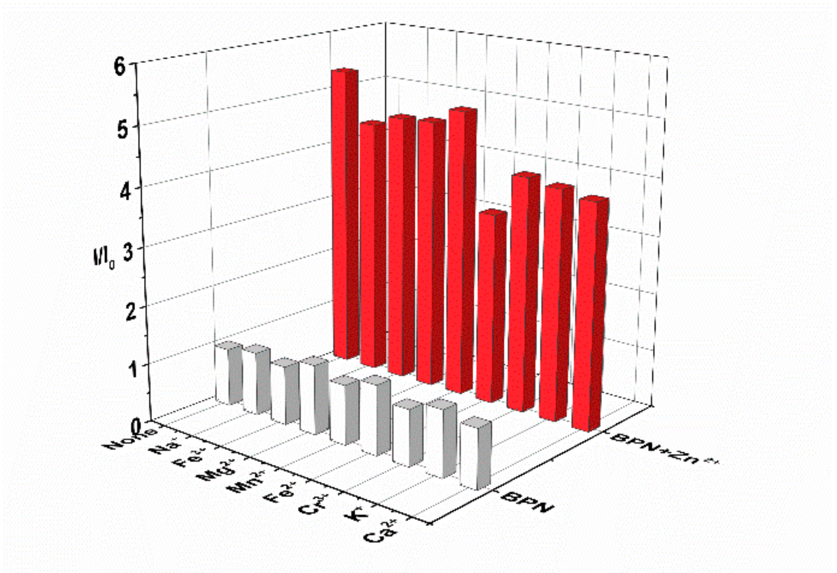

Figure S12 Fluorescence intensity (*I*) responding to other metal ions of nitrates (30 μM) with zinc ions (30 μM) existed or not. I_0 is the fluorescence intensity of **BPN** (30 μM)

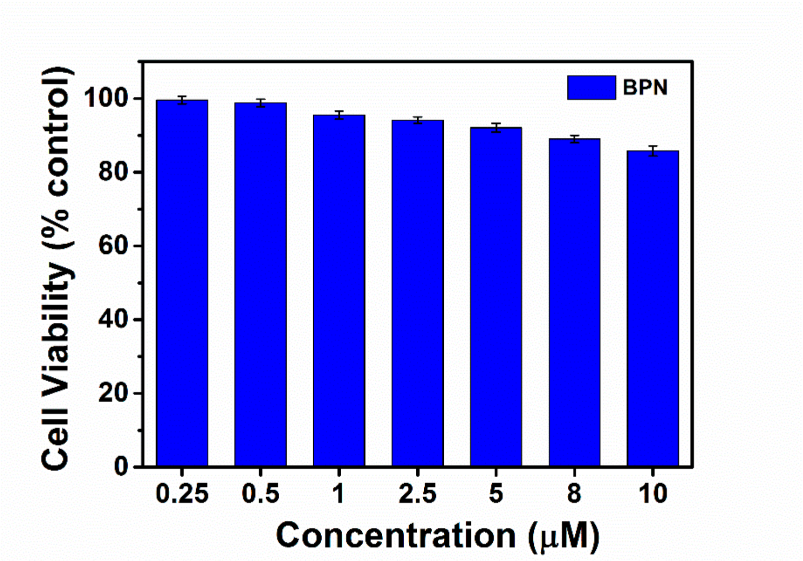

Figure S13 BPN cytotoxicity analyzed by MTT assays at various concentrations (0.25, 0.5, 1, 2.5, 5.0, 8.0, and 10.0 μM) in living MCF-7 cells for 12 h

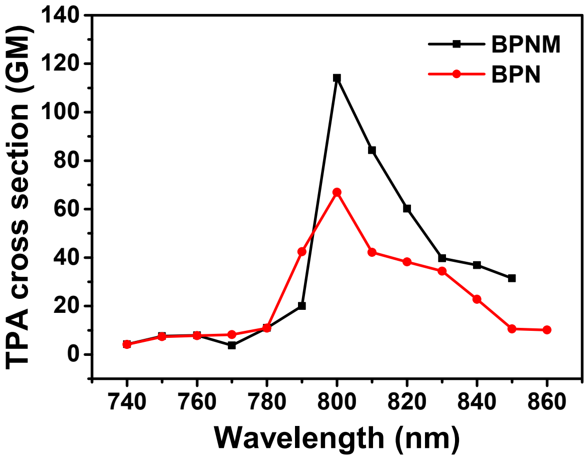

Figure S14 Two-photon action (TPA) spectra of **BPNM** (0.1 μM) and **BPN** (0.1 μM Tris-HCl 10 mM, 60 mM KCl, pH=7.4)
